# Bevacizumab combined with chemotherapy for glioblastoma: a meta-analysis of randomized controlled trials

**DOI:** 10.18632/oncotarget.16924

**Published:** 2017-04-07

**Authors:** Shou-Bo Yang, Kai-Di Gao, Tao Jiang, Shu-Jun Cheng, Wen-Bin Li

**Affiliations:** ^1^ Department of Oncology, Beijing Shijitan Hospital, Capital Medical University, Beijing, China; ^2^ Beijing Rehabilitation Hospital of Capital Medical University, Beijing, China; ^3^ Department of Neurosurgery, Tiantan Hospital, Capital Medical University, Beijing, China

**Keywords:** bevacizumab, chemotherapy, glioblastoma, meta-analysis, prognosis

## Abstract

Bevacizumab, as antibodies, were applied to inhibit tumor angiogenesis by preventing activation of vascular endothelial growth factor receptor. We analyzed four clinical trials, including 607 patients, to investigate the efficacy and safety of bevacizumab when combined with chemotherapy for the treatment of glioblastomas. Results demonstrated that bevacizumab when combined with chemotherapy improved progression-free survival (HR = 0.66; 95% CI 0.56–0.78; *p* < 0.00001) compared with bevacizumab or chemotherapy alone. Furthermore, overall survival showed insignificant difference between two arms (HR 0.99; 95% CI 0.8–1.21; *p* = 0.92). However, we found that patients treated with bevacizumab-containing therapy reported increased objective response rate (OR 1.85, 95% CI 1.17–2.93; *p* = 0.009), but more treatment-related adverse events (OR 1.75; 95% CI 1.09–2.83; *p* = 0.02).

## INTRODUCTION

Glioblastoma (GBM, WHO grade IV) is the most common brain tumor, accounting for 15.1% of all primary brain tumors and 46.1% of all malignant brain tumors. The assessed number of cases of GBMs in the United States for 2015 and 2016 are 11,890 and 12,120, respectively [1]. For newly diagnosed GBMs, the regimen developed by Professor Stupp is considered as standard therapy, comprising maximal resection, radiochemotherapy, and temozolomide maintenance therapy [2]. However, GBM is typical of aggressiveness and always indicate a poor prognosis. The median survival rate of GBM patients is approximately one year, and only 5.1% can survive five years post diagnosis [1].

GBMs are composed of densed and highly disorganized vessels [3, 4]. These highly disorganized vessel architectures critically contribute to rapid tumor growth and resistance to radiotherapy and chemotherapy. Tumor angiogenesis depends on multiple mechanisms. Vascular endothelial growth factor (VEGF) is a crucial factor in these signal passways affecting angiogenesis [5]. GBM cells present a high level of VEGFs and were correlated to aggressiveness and prognosis [6]. Thus, the inhibition of VEGF is assumed to slow down tumor growth and enhance the effects of radiotherapy and chemotherapy [7, 8].

Bevacizumab is a monoclonal antibody that targets VEGF-A to inhibit its downstream signal activity by restraining interaction with the VEGF receptor. It was the first drug targeting tumor angiogenesis approved by the FDA and was applied for the treatment of GBMs since 2009 [9]. Various Phase II clinical trials with bevacizumab single-agent therapy showed promising results in recurrent GBMs with progression-free survival (PFS) at six months of 29%–46% [10–12]. However, further studies found no improved overall survival (OS) or relatively high response rate [13]. Researchers implied that bevacizumab inhibits tumor momentarily, but promote tumor growth in the long run [14–16]. The combination therapy with bevacizumab and other chemotherapies was supposed to be more effective. Hence, several trials have been initiated to explore the efficacy of combination therapy.

In this meta-analysis, we aimed to collect data from high-quality randomized controlled trials to assess the efficacy and safety of bevacizumab combined with chemotherapy versus single-agent therapy for new diagnosed and recurrent GBMs.

## RESULTS

### Study selection

**Figure 1:**
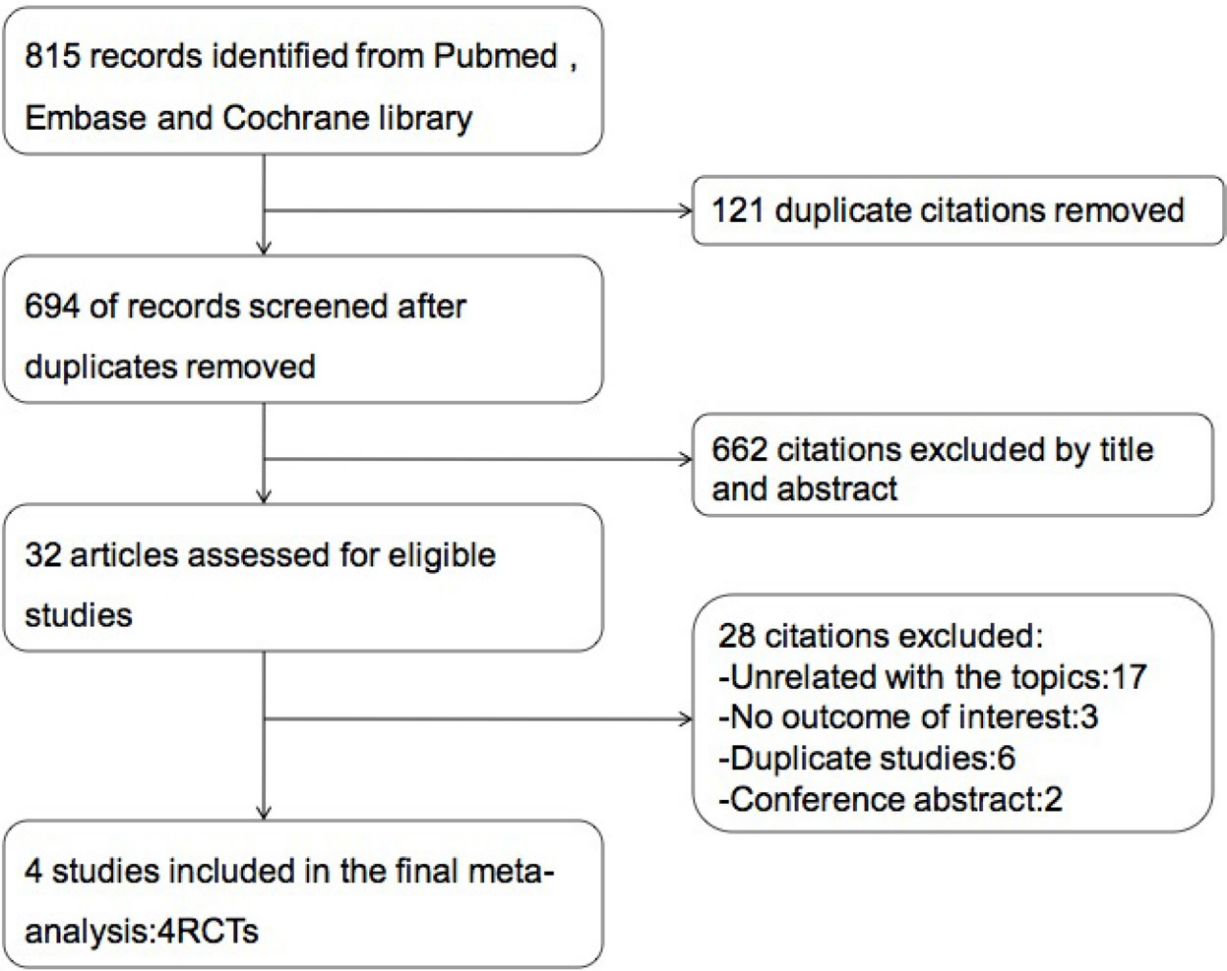
Flow chart of the literature research

**Figure 2:**
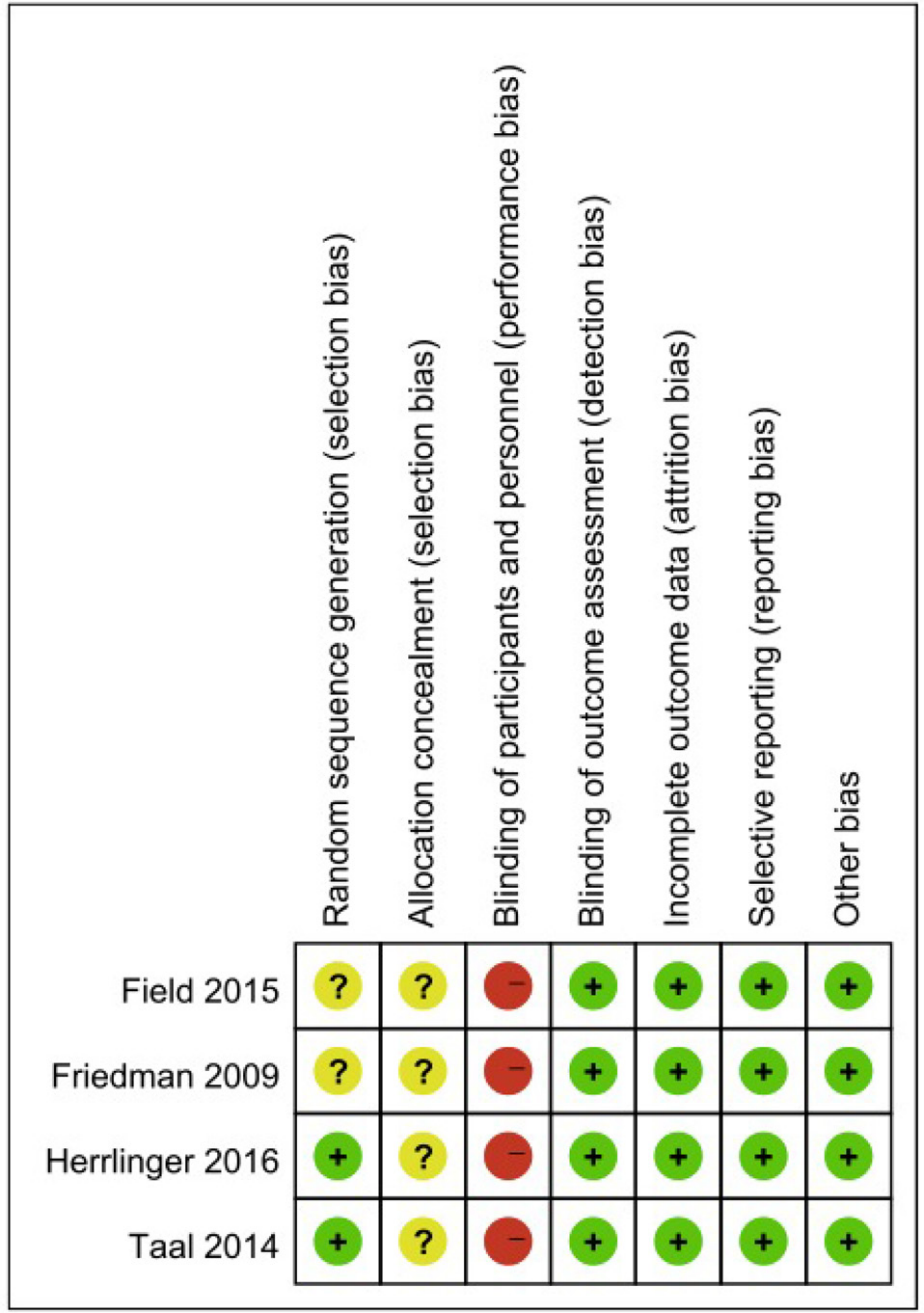
Risk of bias summary Green circle, low risk of bias; Yellow circle, middle risk of bias; Red circle, high risk of bias.

A total of 815 articles were identified by searching database (110 studies from Pubmed, 679 from Embase and 26 from Cochrane library). The identification of the four studies included as shown by a flow chart in Figure 1. The risk of bias for each eligible study is summarized in Figure 2. Four trials were open-labeled and deprived information about allocation concealment [20–23]. Two trials deprived information about random sequence generation [20, 23].

### Characteristics of the included studies

The details of baseline characteristics in five included studies are shown in Table 1. A total of 607 patients were enrolled in these multicenter studies (310 patients randomized to bevacizumab combination therapy group and 297 to control group). All patients were above 18 years old and histologically confirmed GBMs. Four studies were multicenter, unblended and phase II randomized clinical trials. Three trials focused on recurrent GBMs, and one other trial focused on newly diagnosed GBMs. The experimental group regimens were bevacizumab combined with irinotecan, carboplatin and lomustine, whereas the control group regimens were bevacizumab monotherapy or chemotherapy alone, correspondingly [20–23].

**Table 1:** Baseline characteristics of the patients in the trials included in the meta-analysis

Study	Recruitment period	*N*	Number of men	Mean age	First-line therapy	Intervention		Median PFS (months)		Median OS (months)		ORR		AES(Grade ≥ 3)	
						Exp	Con	Exp	Con	Exp	Con	Exp	Con	Exp	Con
**Field 2015**	2010–2012	122	67 (55%)	55	No	BEV + CAR	BEV	3.5	3.5	6.9	7.5	8 (14%)	4 (6%)	37 (64%)	36 (58%)
**Friedman 2009**	2006–2007	167	115 (69%)	55	No	BEV + IRI	BEV	5.6	4.2	9.2	8.7	31 (37.8%)	24 (28.2%)	52 (66%)	39 (46%)
**Herrlinger 2016**	2010–2012	170	114 (67%)	56	Yes	BEV + IRI	TMZ	9.7	5.99	16.6	17.5	NA	NA	NA	NA
**Taal 2014**	2009–2011	148	91 (61%)	57	No	BEV + LOM	BEV/LOM	4	3/1	12	8/8	19 (39%)	20 (22%)	NA	NA

PFS, progression-free survival; OS, overall survival; ORR, object response rate; AES, adverse events; Exp, experimental group; Con, control group; BEV, bevacizumab; CAR, carboplatin; IRI, iriontecan; TMZ, temozolomide; LOM, lomustin.; NA, not available.

### PFS

**Figure 3:**
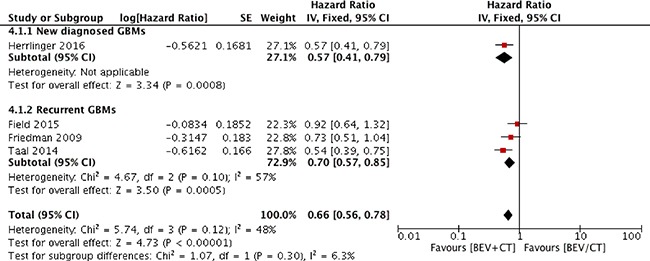
Progression-free survival for combination therapy of bevacizumab plus chemotherapy versus bevacizumab or chemotherapy alone

Three trials reported prolonged median PFS from experimental group to control group, other than one trial with same PFSs in both arms as presented in Table 1 [20–23]. Figure 3 shows the meta-analysis results that the combination of bevacizumab with chemotherapy significantly improved PFS compared with bevacizumab or chemotherapy alone (HR 0.66; 95% CI 0.56–0.78; *p* < 0.00001). Heterogeneity among the trials was not statistically significant (χ^2^ = 5.74; *p* = 0.12; I^2^ = 48%).

A subgroup analysis based on the line of treatment was also performed. The results indicated that bevacizumab combined with chemotherapy prolonged PFS in recurrent GBM patients (HR 0.70; 95% CI 0.57–0.85; *p* = 0.0005; Figure 3).

### OS

**Figure 4:**
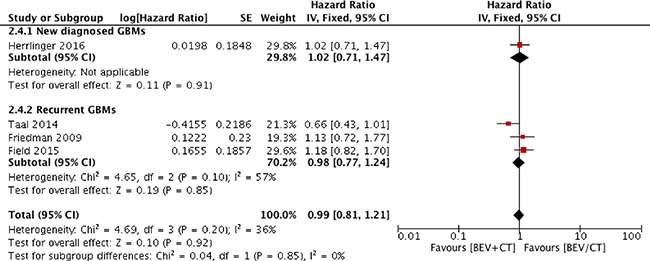
Overall survival for combination therapy of bevacizumab plus chemotherapy versus bevacizumab or chemotherapy alone

All four trials demonstrated insignificant statistical differences between experimental group and control group (Table 1) [20–23]. In the meta-analysis of the four trials, results from Figure 4 suggested that bevacizumab combined with chemotherapy was not associated with any significant improvement in OS (HR 0.99; 95% CI 0.81–1.21; *p* = 0.92). Insignificant heterogeneity was found in pooled trials (χ^2^ = 4.69; *p* = 0.20; I^2^ = 36%).

The subgroup analysis outcomes made no difference with overall results above. There were insignificant statistical differences in recurrent GBM patients (HR 0.98; 95% CI 0.77–1.24; *p* = 0.85; Figure 4).

### ORR

**Figure 5:**
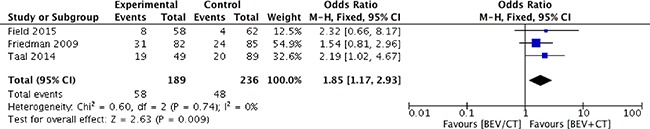
Object response rate for combination therapy of bevacizumab plus chemotherapy versus bevacizumab or chemotherapy alone

Three trials offered ORR values (including complete response and partial response), as shown in Table 1 [20, 22, 23]. As Figure 5 presents, pooled values were analyzed and established that bevacizumab combined with chemotherapy was associated with a significantly better ORR (OR 1.85; 95% CI 1.17–2.93; *p* = 0.009). However, no heterogeneity was identified among the pooled studies (χ2 = 0.60; *p* = 0.74; I^2^ = 0%).

### AES

**Figure 6:**
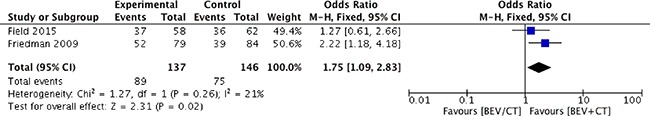
Adverse events for combination therapy of bevacizumab plus chemotherapy versus bevacizumab or chemotherapy alone

Of the included studies, two reported the data of adverse events [20, 23]. Adverse events of grade 3/4 listed in Table 1 were compared. As Figure 6 shows, the pooled estimates suggested that the combination therapy of bevacizumab and chemotherapy induced a significantly higher rate of high grade AES (OR 1.75; 95% CI 1.09–2.83; *p* = 0.02) compared with bevacizumab or chemotherapy alone. However, no heterogeneity was identified among the pooled studies (χ^2^ = 1.27; *p* = 0.26; I^2^ = 21%).

## DISCUSSION

This study was a meta-analysis of RCTs and compares the combination of bevacizumab and chemotherapy to bevacizumab or chemotherapy alone in the treatment on GBMs. Our results show that, compared with a single-agent therapy, a combination treatment with bevacizumab and chemotherapy can yield improved PFS but not better OS in GBMs. A subgroup analysis in recurrent GBMs was also performed that resulted in the same conclusion. Additionally, compared with control group regimens, the combination regimen offers a greater ORR but more frequent grade 3/4 AES.

An article on meta-analysis of bevacizumab plus temozolomide- radiotherapy for newly diagnosed glioblastoma with different O^6^-methylguanine–DNA methyltransferase (MGMT) methylation status was published [24]. In that study, three clinical trials were included to assess the treatment efficacy. Their results presented that, compared with temozolomide and radiotherapy, bevacizumab combined with temozolomide and radiotherapy could improve PFS significantly in both MGMT methylated and unmethylated patients (pooled HRs, 0.77, *p* = 0.032; 0.68, *p* = 0.038). However, the combination treatment could not prolong OS significantly (pooled HRs, 1.132, *p* = 0.345; 1.018, *p* = 0.345). Their conclusion was consistent with ours, in which PFS, but not OS, was improved compared with bevacizumab with chemotherapy to bevacizumab or chemotherapy alone. PENG FU reported a meta-analysis including the same three clinical trials with the study above and drew similar results of PFS (HR 0.71; 95% CI 0.58–0.87; *p* = 0.001) and OS (HR 0.99; 95% CI 0.77–1.26; *p* = 0.04) [25]. Furthermore, ORR and AES were analyzed by ORs to estimate the combination therapy of bevacizumab with chemotherapy.

Recurrent GBMs used irinotecan as a topoisomerase I inhibitor (PFS at 6 months 16%) [26]. In a previous phase II trial of recurrent GBMs, bevacizumab in combination with irinotecan increased 6-month PFS to 46% [27]. In our pooled studies, Friedman reported a phase II, multicenter, open-label, non-comparative trial to estimate the efficacy of bevacizumab plus irinotecan for recurrent GBMs [20]. Compared with bevacizumab monotherapy, the combination treatment increased PFS-6 from 42.6% to 50.3% and ORR from 28.2% to 37.8%. Median PFS was prolonged from 4.2 months to 5.6 months, but the PFS difference did not attain significance, calculated by the Kaplan–Meier curve (HR 0.73; 95% CI 0.51–1.04; *p* = 0.085). However, the OS benefit was not improved (median values from 9.2 months to 8.7 months) (HR 1.13; 95% CI 0.72–1.77; *p* = 0.60). Serious AES (grade 3/4) was experienced by 66% and 46% of patients in the combination and monotherapy group, respectively. Another phase II unblended trial assessed the efficacy of bevacizumab combined with irinotecan for newly diagnosed O^6^-methylguanine–DNA methyltransferase nonmethylated GBMs [21]. In this trial, control group regimen was temozolomide monotherapy. PFS-6 was increased from 42.6% with temozolomide to 79.3% with BEV+IRI (*p* < 0.001). The median PFS was prolonged from 5.99 months to 9.7 months (95% CI, 8.7–10.8 months; *p* < 0.001). No statistical difference of OS was observed between the groups: the median OS was 17.5 months with temozolomide; and 16.6 months with bevacizumab plus irinotecan.

Carboplatin and lomustine were traditionally used cytotoxic drugs for GBMs, with typical response rates less than 20%; PFS-6 has a response rate of around 15%; OS is generally less than 6 months [28–30]. These two cytotoxic drugs have been involved with two trials in this meta-analysis. In a multicenter, randomized phase II trial, researchers explored the combination treatment of bevacizumab with carboplatin for recurrent GBMs [23]. That trial gained the same median PFS of 3.5 months for both groups (HR 0.92; *p* = 0.66). OS between the arms also showed insignificant difference (HR 1.18; *p* = 0.38). AES in the trial was also recorded. The most frequent AES in the combination group were hematologic adverse events. Data for grade 3/4 AES with 64% in combination group and 58% in control group (*p* = 0.52) were collected. BELOB trial studied the efficacy of bevacizumab plus lomustine in patients with recurrent GBMs [22]. The open-label, multicenter phase II study assigned eligible patients to three groups of bevacizumab or lomustine and bevacizumab and lomustine alone. The trial showed prolonged median PFSs with 1, 3, and 4 months in lomustine, bevacizumab, and bevacizumab or lomustine arm, respectively. Median OS was prolonged with 8, 8, and 12 months, respectively. However, difference of OS did not reach significance. As a method depicted above, the merged HR was calculated to represent outcomes with PFS (HR 0.54; *p* = 0.0004) and OS (HR 0.66; *p* = 0.06) [17].

Bevacizumab was applied for recurrent GBMs due to a high ORR. ORR was thought to be correlated with prognosis of recurrent GBMs by several past studies [31, 32]. In BELOB trial, a trend was found and suggested that a higher ORR was associated with improved OS [22]. As Figure 5 presents, an analysis of ORR by data of three trials was performed to find that the bevacizumab-containing group made better ORR than the monotherapy group [20, 22, 23]. However, prolonged OS was unproven. Different outcomes of PFS and OS are due to the fact that bevacizumab is a two-edged sword. After temporal anti-tumor effects with bevacizumab treatment, sequential hypoxia in GBM microenvironment may enhance invasion of GBM cells [33–36].

This study has some advantages. The four RCTs included were multicenter, large-scale, phase II trials that were well designed and well-performed [20–23]. The assessment of efficacy and safety with bevacizumab was assured to be of high reliability.

Consequently, this meta-analysis has several disadvantages worth noting. First, the number of pooled trials was relatively small; publication bias cannot be excluded. Second, patients in control groups received different treatment regimens: bevacizumab in two trials, temozolomide in one trial, and bevacizumab/lomustine in one trial [20–23]. These different treatment regimens may have an influence on estimated bevacizumab combination therapy. Third, an analysis was performed on overall grade 3/4 AES and subgroup analysis for recurrent GBMs because of insufficient data. Therefore, we look forward to more large-scale phase III trials to be conducted and enough information to perform a more powerful meta-analysis. However, these four eligible trials are still reliable, and our results will be helpful for physicians to make better decisions with bevacizumab treatment.

## MATERIALS AND METHODS

### Search strategy

This meta-analysis was registered at International Prospective Register of Systematic Reviews (number CRD42016047227) and was reported to adopt the Preferred Reporting Items for Systematic Reviews and Meta-Analyses (PRISMA) guidelines.

Relevant studies published in English, from January 1, 2009 to September 1, 2016, were selected by searching PubMed, Embase, and Cochrane. The following medical subject heading (MeSH) terms were used for PubMed: ((Glioblastoma OR Glioma) OR (glioma* OR glioblastoma* OR glioblastoma multiforme) OR Astrocytoma*)) AND (Bevacizumab OR avastin) AND (randomized controlled trial OR controlled clinical trial OR randomized OR placebo OR clinical trials as topic OR randomly OR trial) NOT (animals NOT humans). The reference list of key articles was manually checked to prevent relevant articles from being excluded. Searching potential literature was done by two reviewers (Shou-Bo Yang and Kai-Di Gao), independently.

### Inclusion and exclusion criteria

Studies were selected by the following criteria: (1) subjects were randomized control trials; (2) subjects are adults diagnosed with glioblastoma, irrespective whether primary or recurrent type; and (3) the two arms were treated with bevacizumab plus chemotherapy, bevacizumab, or chemotherapy alone. Furthermore, studies should not satisfy any exclusion criteria: (1) total subjects number was less than 30; and (2) the intervention of control arm was not chemotherapy or bevacizumab, but placebo.

### Data extraction and outcomes of interest

The titles and abstract of all the studies identified in the literature search were screened by two reviewers (Shou-Bo Yang and Kai-Di Gao) to verify its compliance with the inclusion and exclusion criteria. The same reviewers independently extracted the data and assessed the quality of the publications. Disagreements between the two reviewers were resolved by consensus after a joint second review.

Information from included trials was extracted as follows: publication reference; recruitment period; patient characteristics; intervention regimens; number of events; and survival.

The PFS and OS rate were the primary outcome, whereas the object response rate (ORR) and treatment-related adverse events (AES) were the secondary outcome. ORR included complete responses (CRs) and partial responses (PRs). The authors were consulted for additional information for data excluded in the articles.

### Quality assessment

The Cochrane Collaboration’s tool was used to assess the risks of selection, performance, detection, attrition, and reporting biases in the RCTs selected for analysis. Trials with high-risk components of more than two were deemed to have a moderate risk of bias, whereas trials with high-risk components of more than four were deemed to have a high risk of bias.

### Data synthesis and analysis

The time-to-event variables (OS and PFS) were assessed by hazard ratios (HRs) with 95% confidence intervals (CIs) for each study, and the dichotomous variables (ORR and AES) by ORs with 95% CIs. In studies without direct HRs, Kaplan–Meier curves and follow-up period were used to calculate HRs [17]. One study assigned three groups and used the above-mentioned methods to calculate a merged HR with 95% CI of the experimental group versus two control groups. A *p* value less than 0.05 was considered statistically significant.

I^2^ testing was performed to assess the heterogeneity between studies, with values greater than 50% indicating significant heterogeneity. When significant heterogeneity was found, a random-effect model was used to analyze pooled studies; otherwise, a fixed-effects model was applied [18, 19]. As the number of pooled studies was less than 10, publication bias was not assessed. Review Manager 5.3 (Cochrane Collaboration) was used for all statistical analyses.

## CONCLUSIONS

Our meta-analysis suggested that the combination of bevacizumab and chemotherapy can improve PFS and ORR, significantly, but do not prolong OS. Moreover, combination treatment results in a more tolerable frequent grade 3/4 AES. Further studies focusing on the optimal regimen with bevacizumab are needed obtain best benefits to GBM patients.
